# Mechanisms of Resistance in Gram-Negative Urinary Pathogens: From Country-Specific Molecular Insights to Global Clinical Relevance

**DOI:** 10.3390/diagnostics11050800

**Published:** 2021-04-28

**Authors:** Branka Bedenić, Tomislav Meštrović

**Affiliations:** 1 Department of Clinical and Molecular Microbiology, University Hospital Centre Zagreb, 10 000 Zagreb, Croatia; branka.bedenic@kbc-zagreb.hr or; 2 Department of Medical Microbiology and Parasitology, School of Medicine, University of Zagreb, 10 000 Zagreb, Croatia; 3 Clinical Microbiology and Parasitology Unit, Dr. Zora Profozić Polyclinic, 10 000 Zagreb, Croatia; 4 University Centre Varaždin, University North, 42 000 Varaždin, Croatia

**Keywords:** urinary tract infections, UTI, gram-negative bacteria, antibiotics, antimicrobial drugs, resistance mechanisms, molecular diagnostics, microbiology

## Abstract

Urinary tract infections (UTIs) are the most frequent hospital infections and among the most commonly observed community acquired infections. Alongside their clinical importance, they are notorious because the pathogens that cause them are prone to acquiring various resistance determinants, including extended-spectrum beta-lactamases (ESBL); plasmid-encoded AmpC β-lactamases (p-AmpC); carbapenemases belonging to class A, B, and D; *qnr* genes encoding reduced susceptibility to fluoroquinolones; as well as genes encoding enzymes that hydrolyse aminoglycosides. In *Escherichia coli* and *Klebsiella pneumoniae*, the dominant resistance mechanisms are ESBLs belonging to the CTX-M, TEM, and SHV families; p-AmpC; and (more recently) carbapenemases belonging to classes A, B, and D. Urinary *Pseudomonas aeruginosa* isolates harbour metallo-beta-lactamases (MBLs) and ESBLs belonging to PER and GES families, while carbapenemases of class D are found in urinary *Acinetobacter baumannii* isolates. The identification of resistance mechanisms in routine diagnostic practice is primarily based on phenotypic tests for the detection of beta-lactamases, such as the double-disk synergy test or Hodge test, while polymerase chain reaction (PCR) for the detection of resistance genes is mostly pursued in reference laboratories for research purposes. As the emergence of drug-resistant bacterial strains poses serious challenges in the management of UTIs, this review aimed to appraise mechanisms of resistance in relevant Gram-negative urinary pathogens, to provide a detailed map of resistance determinants in Croatia and the world, and to discuss the implications of these resistance traits on diagnostic approaches. We summarized a sundry of different resistance mechanisms among urinary isolates and showed how their prevalence highly depends on the local epidemiological context, highlighting the need for tailored interventions in the field of antimicrobial stewardship.

## 1. Urinary Tract Infections: Introduction, Epidemiology, Pathophysiology, and Pathogen Profile

Urinary tract infections (UTIs) are the most frequent hospital infections, and second among community acquired infections after respiratory tract infections, affecting hundreds of millions of individuals every year [1]. These infections can be distressing and, in certain instances, even life threatening, with both females (40%) and males (12%) reporting at least one symptomatic urinary tract infection (UTI) during their lives [2]. UTIs develop as a result of the presence and multiplication of microorganisms in the different parts of the urinary system, subsequently resulting in tissue invasion, inflammatory response, and various symptoms depending on the localization of the infection [3]. Urine is considered a primary sterile body fluid; however, it can easily get contaminated with microorganisms stemming from the urethra, perineum, or vagina, while newer studies challenge this paradigm as the presence of a urinary microbiome is being recognized [4]. The protection against UTI is based on normal urine flow, low pH, and high osmolality preventing replication of potential pathogens that can cause disease [3]. Intrinsic protective factors include mucus immunoglobulin A (IgA) antibodies, cytokine, and chemokine production [5].

Risk factors for the development of UTI comprise gender, age, the presence of urinary catheters, pregnancy, vesicoureteral reflux, urinary incontinence, immunosuppression, hospitalization, and organ transplantation [6,7]. Uropathogenic bacteria possess a plethora of virulence factors involved in the pathogenesis (type I pili, type III pili, alginate, haemolysins, aerobactin, siderophores), but they primarily utilize fimbriae to mediate the attachment to the urinary epithelium and subsequent penetration [3,7]. Following this initial step, the pathogens can migrate to the kidney (by using flagella) and cause pyelonephritis [8]. In the outpatient setting, UTIs usually arise due to the ascendant spread of endogenic microbiota from the intestines. Consequently, among adults, they are more frequently observed among woman due to shorter urethra, whereas in men UTIs are usually considered complicated and linked to prostatic hypertrophy or adenoma [8]. Conversely, UTIs in the hospital setting are usually associated with urinary catheters, with the source of infection being intestinal microbiota or contaminated hands of the hospital staff [9]. In older populations urinary catheters are important risk factors for UTIs, particularly in long-term care facilities [9]. UTIs in children are most frequently associated with urinary tract abnormalities such as vesicoureteral reflux [10]. According to the guidelines, UTIs can be classified as acute uncomplicated cystitis in woman, acute uncomplicated pyelonephritis, complicated urinary tract infections in men, asymptomatic bacteriuria, and recurrent UTIs [9]. 

Acute UTIs are associated with a large number of bacterial agents (>10^5^ colony-forming units per millilitre or CFU/mL), whereas lower numbers are usually due to contamination with intestinal microbiota [11]. For that reason, urine culture is pursued as a quantitative method based on determination of bacterial count, and the role of the clinical microbiologist is to distinguish between true bacteriuria and contamination. According to the current guidelines [9,12], in acute, uncomplicated cystitis, urine culture is not necessary, as the diagnosis is established on the basis of urine sediment examination or a leukocyte esterase test. Acute uncomplicated pyelonephritis is confirmed by urine culture (≥10^4^ CFU/mL in pure culture) and urinary sediment analysis. Complicated urinary tract infections are diagnosed based on leukocyte esterase and urine culture with ≥ 10^4^ CFU/mL, except in pregnancy when the lower CFU is also considered significant (≥10^3^ CFU/mL) [12]. Asymptomatic bacteriuria in women is characterized by ≥ 10^5^ CFU/mL in pure culture in two consecutive midstream urine cultures taken 24 h apart; conversely, in men it is sufficient to obtain one urine culture. In recurrent cystitis the breakpoint is ≥ 10^3^ CFU/mL in pure culture, whereas in pyelonephritis it is ≥10^4^ CFU/mL [9,12]. 

Primary urinary pathogens are *Escherichia coli* (*E. coli*) and *Staphylococcus saprophyticus* (*S. saprophyticus*), responsible for 80% of UTIs in healthy individuals [13,14]. Secondary pathogens are principally Gram-negative species such as *Enterobacter* spp., *Klebsiella* spp., *Proteus mirabilis* (*P. mirabilis*), *Morganella morganii* (*M. morganii*), *Citrobacter* spp., *Serratia* spp., *Pseudomonas aeruginosa* (*P. aeruginosa*), and *Acinetobacter baumannii* (*A. baumannii*) [9,13,14]. Among Gram-positive species, *Enterococcus* spp. and *Stapyhlococcus aureus* are categorized as secondary pathogens [9,15]. The whole group of secondary pathogens is rarely observed in uncomplicated UTIs, but are frequently isolated in complicated urinary tract infections, as well as from hospitalized patients [16]. For *Streptococcus agalactiae* (*S. agalactiae*), *Stenotrophomonas maltophilia*, *Burkholderia cepacia*, and yeasts, there is no adequate evidence at the moment regarding their pathogenicity, albeit they are potentially deemed important when isolated in large numbers and in repeated urine samples. Furthermore, *S. agalactiae* can be considered an important pathogen in pregnant woman and patients suffering from diabetes mellitus, while alpha-haemolytic streptococci, lactobacilli, diphtheroids, and *Gardnerella vaginalis* belong to the urogenital microbiota [4,15].

Nonetheless, the emergence of drug-resistant bacterial strains in the management of UTIs represents one of the most pertinent public health issues of the modern era [1]. This review aims to appraise mechanisms of resistance in Gram-negative urinary pathogens, provide a detailed map of resistance determinants in Croatia and put it within the global context for all relevant Gram-negative pathogens, and to discuss the implications of these resistance traits on clinical approaches and the choice of antimicrobial drugs.

## 2. The Evolving Story of Resistance Determinants

As already mentioned, antimicrobial resistance in the urinary pathogens is increasingly being observed, primarily as a result of our choices regarding antimicrobial treatment. It commonly occurs as a result of target alteration, diminished drug accumulation, and drug modification [14]. Accordingly, the most pertinent resistance determinants found among urinary tract pathogens are extended-spectrum beta-lactamases (ESBL), plasmid-mediated AmpC beta-lactamases, carbapenemases, and reduced susceptibility to fluoroquinolones due to the acquisition of *qnr* genes [7,14]. 

The first extended-spectrum beta-lactamase (ESBL) was SHV-2 described in Germany in 1983 in *Klebsiella oxytoca* [17]. Following that discovery, the spread of ESBL-positive *Enterobacteriaceae* initially occurred throughout Europe and later all over the world. Today, they are the most frequent among hospital isolates of *K. pneumoniae* and *E. coli*, but they have also been reported among community isolates of *Enterobacteriaceae* [18]. The majority of ESBLs belong to three families: TEM (Temoneira), SHV (sulfhydryl variable), and CTX-M (cefotaximase). TEM and SHV variants are dominant among hospital isolates, while CTX-M variants are typical for community isolates [14]. Moreover, TEM and SHV variants are derived by mutations of parental broad spectrum TEM-1, TEM-2, and SHV-1 beta-lactamases [18]. CTX-M beta-lactamases are a growing family of plasmid-encoded ESBLs that preferentially hydrolyse the antimicrobial agent cefotaxime. They are not closely related to TEM or SHV beta-lactamases, but they are typical members of Ambler’s class A derived from the Gram-negative bacterial genus *Kluyvera* [19].

In contrast to TEM and SHV beta-lactamases, which rely on amino acid substitutions to extend their substrate profile, CTX-M enzymes have an intrinsic extended-spectrum profile [20]. Furthermore, contrary to TEM and SHV enzymes, which are usually associated with hospital pathogens, CTX-M are predominantly isolated from community acquired infections [21]. CTX-M beta-lactamases are the dominant type of ESBLs in many countries of the world such as the United Kingdom, Italy, Spain, Greece, Poland, Bulgaria, Russia, Latvia, Brazil, China, Taiwan, and many others [21,22,23]. There are also rare types of ESBLs such as VEB, PER, or IBC [18]. Genes encoding ESBLs are almost always located on a transferable plasmid, which often contains genes for aminoglycoside, tetracycline, sulphonamide, and fluoroquinolone resistance [18]. Furthermore, ESBL-producing *Enterobacteriaceae* strains can cause outbreaks of nosocomial infections [24,25,26], which are rather difficult to control due to their multiresistant phenotype [27].

Plasmid-mediated AmpC beta-lactamases are derived from the chromosomal beta-lactamases of the bacteria belonging to the genera *Enterobacter*, *Serratia*, *Citrobacter*, *Pseudomonas,* and *Acinetobacter* by the escape of the chromosomal gene to a plasmid [14,19,28]. They hydrolyse third generation cephalosporins, monobactams, and cephamycins but spare fourth generation cephalosporins and carbapenems. Unlike ESBLs, they are not susceptible to inhibition with clavulanic acid, sulbactam, or tazobactam [19,28]. 

Carbapenems are often considered as “the last resort antibiotics” for the treatment of severe infections associated with ESBLs or AmpC-producing Gram-negative bacterial agents. Acquired resistance to carbapenems was not commonly observed until recently [29]. Beta-lactamase-mediated resistance to carbapenems is mostly due to the expression of class A KPC beta-lactamases, susceptible to the inhibition by clavulanic acid, class B metallo-beta-lactamases of the IMP, VIM, or NDM series, or OXA-48 beta-lactamase belonging to the class D beta-lactamases that are not inhibited by clavulanic acid, tazobactam, and sulbactam and thus confer on the producing isolates resistance to beta-lactam inhibitor combinations [30,31].

## 3. *Escherichia coli*

*E. coli* is the most frequent urinary pathogen in the outpatient setting [3,5,8]. Around 70% of the isolates are resistant to ampicillin due to the production of TEM-1 broad spectrum beta-lactamase [5]. Furthermore, the resistance to expanded-spectrum cephalosporins is mainly attributed to the production of ESBLs belonging to TEM, SHV, and CTX-M families, and to a lesser extent mediated by plasmid encoded AmpC beta-lactamases [32]. ESBL rates in *E. coli* range from around 4% in Europe, 24% in Africa, to 50% in Nepal [33,34]. TEM and SHV variants were dominant in the 1990s, whereas CTX-M have been increasing recently all over the world, with the CTX-M-15 variant being widespread around the globe with a resistance phenotype including a high level of resistance to extended-spectrum cephalosporins (ESCs) and ciprofloxacin [35]. There are many reports on this allelic variant in urinary isolates from South America [36,37], Africa [38], and Asia [34]. Other CTX-M allelic variants found among urinary isolates were CTX-M-1, CTX-M-3, CTX-M-14, and CTX-M-27 [39]. Some other ESBLs found in urinary *E. coli* isolates are SHV-7 and OXA-1 [33]. In Africa, TEM-22 outnumbered CTX-M variants among urinary *E. coli* isolates [33]. The rate of AmpC beta-lactamases reaches 30% in some geographic areas, with CMY-2 type being the most prevalent [33].

Moreover, a study from Iraq found a prevalence of ESBLs of 7% among *E. coli* associated with uncomplicated UTIs of the lower urinary tract, while in the upper urinary tract infections the prevalence was 30% [40]. In that study, TEM-ESBLs were dominant in both types of infections with rates of 31% and 65%, respectively, followed by CTX-M β-lactamases (24% vs. 60%), whereas SHV was the least prevalent (28% vs. 50%) [40]. A recent study conducted in Romania demonstrated high resistance rates among uropathogenic *E. coli* with resistance rates of 48% for ampicillin, 41% for tetracycline, 24% for cotrimoxazole, 19% for amoxicillin-clavulanic acid, 16% for cefazolin, 15% for ciprofloxacin, and 15% for levofloxacin; furthermore, 35% of the investigated strains were multidrug resistant [41]. This report identified 9.3% of the uropathogenic isolates as harbouring ESBLs with 42% being positive for CTX-M beta-lactamases, 38% for TEM, and 19.7% for SHV. The majority of isolates possessed various virulence factors such as type 1 fimbriae (93%), haemolysin D (44%), A fimbriae (38%), capsules (32%), S fimbriae (22%), and haemolysin A (12%) [41]. Resistance to fluoroquinoles, as demonstrated in the aforementioned study, is usually attributed to *qnr* genes (*qnrA*, *qnrB*, *qnrS*) that encode Qnr proteins protecting the topoisomerase enzyme. They were found in approximately 30% of isolates in a recent study by Onanuga et al. [33] from Africa. Moreover, gentamicin resistance was found in 44% of the ESBL-positive isolates due to the *aacC2* gene in the same study [33].

In Croatia, CTX-M ESBLs of group 1 were found to be associated with UTIs in different geographic regions after the year 2000 [42,43,44]. CTX-M-15 was the dominant type, albeit CTX-M-3 was also found in some isolates [43]. *bla*_CTX-M_ genes were carried by FIA and L/M plasmids (according to the plasmid incompatibility grouping) in the early 1990s, and SHV-2 and SHV-5 were found in UTI isolates of *E. coli* [45]. They caused urinary tract infections in neonates and young children, and they were associated with specific virulence factors such as resistance to serum bactericidal activity and haemolysin production. 

There is a positive correlation between biofilm production and ESBL positivity [46]. Among urinary isolates there were 14% strong, 17% moderate, and 22% weak biofilm producers [46]. In the past, ESBL-positive isolates were identified predominantly in hospital acquired UTIs, but recently a dramatic increase of ESBL positivity was observed among community isolates as well [47]. Previous exposure to cefuroxime was found to be a risk factor [47]. Due to an increasing trend of ESBL-positivity in *E. coli* and consequential multidrug-resistant phenotypes, guidelines for the management of UTIs were updated. More specifically, fosfomycin and nitrofurantoin are now recommended as first line empirical oral therapy for the treatment of community acquired, uncomplicated UTI [48].

*E. coli* sequence types (STs) are established with the multilocus sequence typing (MLST), and *E. coli* ST-131 containing CTX-M-15 are mostly of serotype O25:H4; interestingly, the latter serotype was found in clinical urinary specimens and in environmental waters in Sweden, indicating that the urinary tract represents an important pollutant factor for wastewater that comes into the aquatic environment [49]. Among clinical urinary isolates, CTX-M-15 was the dominant type, present in 62%, followed by CTX-M-14 and CTX-M-27, found in 8.9% and 6.7% of the isolates, respectively [49]. The majority of the isolates (33%) belonged to widespread ST-131 lineage, whereas 14% belonged to ST-38. Likewise, the B2 phylogenetic group was the most prevalent (44%) [49].

An increasing trend of multidrug-resistance, mainly due to ESBL production, was observed in *E. coli* isolated from pregnant woman with limited therapeutic options [50]. Earlier studies carried out in the 1990s found plasmid-mediated, transferable SHV-5 beta-lactamase conferring high level ceftazidime resistance among urinary *E. coli* isolates from the United Kingdom, and the isolates possessed additional TEM-1 β-lactamase [51]. TEM-28 was reported in urinary tract *E. coli* isolates in a nursing home in California [52]. Among community isolates, TEM-21 and CTX-M-1 were found to be dominant in France [53]. Spanish reports demonstrated CTX-M-15, CTX-M-14, CTX-M-1, and CTX-M-9 to be the most prevalent in urinary ESBL-positive *E. coli* [54,55]. Those studies have also shown that the *bla*_CTX-M-14_ gene was preceded by the IS*Ecp1* insertion sequence, which plays a significant role in its mobilization and acts as a promotor, increasing expression of the gene [54,55]. Moreover, ESBLs have been reported not only in human medicine, but also in animals. More specifically, *E. coli* producing SHV-12 was identified in urine of a dog suffering from recurrent UTIs [56]. 

Inhibitor-resistant TEM (IRT) beta-lactamases were reported among urinary *E. coli* isolates in Spain. They conferred on producing isolates resistance to amoxicillin/clavulanic acid, ampicillin/sulbactam, and to lesser extent piperacillin/tazobactam. IRT-3 was carried on a transferable 45-kb plasmid [57]. Inhibitor-resistant TEM beta-lactamases are dominant among community acquired infections, which are often treated with β-lactam/inhibitor combinations [53,57]. 

Recently, carbapenemase production was reported in urinary isolates of *E. coli* belonging to class A (KPC), class B (VIM, NDM, IMP), and class D (OXA-48), with the latter showing a trend of significant global spread [58]. The production of carbapenemase is usually associated with an extensively drug-resistant phenotype, leaving very few therapeutic options. A recent study from Pakistan showed significant presence of both ESBL and carbapenemase producers, as well as co-existence of ESBL and carbapenemases in the same isolate [59]. In that study, 50% of isolates were resistant to both imipenem and meropenem, with some isolates expressing the *bla*_NDM-1_ gene [59], which indicates a worrying trend.

## 4. *Klebsiella pneumoniae*

Resistance to expanded-spectrum cephalosporins in *K. pneumoniae* is usually mediated by the production of ESBLs belonging to the TEM, SHV, and CTX-M families or plasmid-mediated AmpC beta-lactamases, mainly DHA or FOX [28]. The rate of ESBL production in *K. pneumoniae* is around 40%, depending on the local epidemiology. Very high rates reaching 50% were observed in urinary isolates in Turkey [60]; nonetheless, carbapenems were shown to possess good activity against ESBL producing isolates. The dominant type is CTX-M-15 found in 80% of the isolates in Portugal, while the dominant ST found in urinary isolates was ST-1 [61]. Conversely, in Iran SHV-ESBLs were dominant; furthermore, among ESBL-positive organisms, 10% were hypervirulent, whereas 22% were found to possess *qnrA*, *qnrB,* and *qnrS* genes [62]. However, it has to be emphasized that these studies are from quite different years, which has to be taken into account when discussing their findings.

A recent study carried out in Iraq found that 59% of the isolates obtained from upper urinary tract infections carried *bla*_ESBL_ genes, in contrast to 18% from the lower urinary tract [40]. The rates of TEM, SHV, and CTX-M beta-lactamases in kidney infections were 88%, 76%, and 6.4%, respectively. Moreover, *bla*_TEM_ genes were also dominant in lower urinary tract infections (83%), followed by *bla*_SHV_ (55%) and *bla*_CTX-M_ (6%) [40].

Earlier studies from the early 1990s identified SHV-2 conferring a high level of cefotaxime resistance among urinary isolates in Germany and the US [63,64]. SHV-5 was reported among urinary isolates in the Netherlands in the early 2000s [65]. The isolates expressed AadB and AadA2 aminoglycoside resistance determinants and had the overexpression of multidrug efflux pumps. Hungarian urinary isolates possessed SHV-2a and SHV-5 on conjugative 217-kb plasmids [66]. Furthermore, urinary isolates from Poland were shown to possess TEM-48 [67]. The first report on transferable resistance to extended-spectrum cephalosporins in urinary *K. pneumoniae* isolates originates from 1987 in France [68]. Since molecular identification of resistance genes was not available in this period, the beta-lactamase was designated as TEM-1-like. The outpatient *K. pneumoniae* isolates in France in the early 2000s were positive for TEM-15, TEM-19, TEM-21, TEM-24, and SHV-4 [53]. Rare types of ESBLs such as VEB-1 were demonstrated in the Far East [69]. Aside from ESBLs, plasmid-mediated AmpC beta-lactamase DHA-1, originating from *Morganella morganii*, was responsible for resistance to expanded-spectrum cephalosporins in urinary isolates of *K. pneumoniae* in France [70].

Studies carried out in Croatia demonstrated SHV-2, SHV-2a, and SHV-5 to be associated with UTIs in the 1990s [71,72]. However, a shift to CTX-M of group 1 was demonstrated after 2000, initially within the in-hospital setting [73]. Very soon after that, the dissemination of group 1 CTX-M beta-lactamases was detected among *K. pneumoniae* in the outpatient setting [74]. Recently, carbapenem resistance emerged in *K. pneumoniae* isolates (which include UTI isolates) due to the production of carbapenemases of class A (KPC), class B (VIM, IMP, NDM), and class D (OXA-48); in the last decade, OXA-48 has become a dominant type of carbapenemase in *K. pneumoniae* in Europe and the Middle East [58]. The first carbapenem-resistant urinary isolates of *K. pneumoniae* were recorded in Croatia in 2012, and they were found to produce VIM-2 and NDM-1 [75]. All metallo-beta-lactamase (MBL) isolates showed an extensively drug-resistant (XDR) phenotype, including resistance not only to beta-lactam antibiotics, but also to aminoglycosides and fluoroquinolones. VIM-2 was the dominant carbapenemase among urinary tract isolates in Croatia until 2015 [76], when it was replaced by OXA-48, which rapidly spread in all geographic regions in Croatia and also in other European countries. *bla*_VIM_ genes were carried by A/C and N plasmids, whereas L/M plasmids harboured *bla*_OXA-48_ genes [77]. Although OXA-48 does not hydrolyse extended-spectrum cephalosporins, the majority of isolates harboured additional ESBLs, conferring resistance to cephalosporins as well [77].

## 5. *Pseudomonas aeruginosa*

*P. aeruginosa* is an important causative agent of urinary tract infections in the hospital setting [78]. It possesses multiple intrinsic and acquired resistance mechanisms, including the overexpression of AmpC and the production of ESBLs and carbapenemases, mostly belonging to class B. Carbapenem resistance in *P. aeruginosa* is usually mediated by the production of metallo-beta-lactamases of IMP, VIM, GIM, SPM, or NDM series; loss of OprD outer membrane protein; and/or upregulation of MexAB or MexCD efflux pumps [78]. In Mediterranean countries, VIM-2 is the most frequent carbapenemase found in *P. aeruginosa*, whereas IMP variants predominate in the Far East [79]. Recently, NDM-1 and KPC-2 were found in carbapenem-resistant *P. aeruginosa* [78,79]. ESBLs are rare in *P. aeruginosa*, compared to *Enterobacteriaceae*, and are predominantly of unusual types such as PER, VEB, and IBC families [79]. OXA-type ESBLs were reported among urinary isolates from Egypt, conferring resistance to ceftazidime, cefepime, and aztreonam [80]. Resistance to aminoglycosides is usually due to the production of acetylases, adenylases, and phosphorylases, which modify aminoglycosides and render them inactive, while resistance to fluoroquinolones is mediated by mutations in *parC* and *gyrA* genes [78,80]. 

A recent study conducted in Iran showed that 8% of isolates were multidrug resistant [81]. Among the resistant isolates, 31.2% and 75% were ESBL and MBL producers, respectively. The prevalence of *bla*_OXA10_, *bla*_VIM_, *bla*_OXA48_, *bla*_OXA-2_, and *bla*_CTX-M_ was 100%, 50%, 31.2%, 25%, and 12.5%. [81]. Furthermore, two isolates (12.5%) harbored *bla*_PER_ and *bla*_NDM_ genes. Urinary tract isolates showed high diversity of different pulsotypes. Ceftazidime-avibactam and ceftolozane-tazobactam were shown to express high antimicrobial activity against urinary *P. aeruginosa* isolates with only 8% of resistant isolates, and the isolates resistant to ceftazidime-avibactam and cefotolozane-tazobactam were positive for ESBLs or MBLs [81]. However, PER-1 ESBL was found among urinary *P. aeruginosa* isolates in northern Italy much earlier [82]. The isolates exhibited a multidrug-resistant phenotype, including resistance to expanded-spectrum cephalosporins, aztreonam, meropenem, aminoglycosides, and ciprofloxacin, and were associated with nosocomial outbreaks. PER-1 was chromosomally encoded and was not transferable. SHV-5 was identified in a nosocomial outbreak involving *P. aeruginosa* from various specimens including urine in Athens in Greece [83]. In addition to expanded-spectrum cephalosporins, the isolates were resistant to aminoglycosides and fluoroquinolones.

Studies carried out in Croatia showed the emergence of VIM-2 among urinary tract isolates in 2004–2005 in Zagreb (i.e., the capital of Croatia) [84]. VIM-2 producing organisms displayed a high level of resistance to all beta-lactam antibiotics (except aztreonam), and also to aminoglycosides and fluoroquinolones. At the same time VIM-2-positive urinary *P. aeruginosa* isolates were identified in the southern region of Croatia; they all belonged to ST-111 and were embedded in class 1 integrons, which also carried *aac* genes responsible for aminoglycoside resistance and the *bla*_OXA-1_ gene [79]. The dominant role of OprD loss and efflux pump upregulation in the resistance to carbapenems was demonstrated later, as was the lack of acquired carbapenemases [85].

## 6. *Acinetobacter baumannii*

Carbapenem resistance in *A. baumannii* is due to production of carbapenemases of class D (CHDL), class B, or rarely class A; moreover, porin loss or upregulation of efflux pumps can contribute to the resistance phenotype [86]. Molecular analysis of carbapenemases in urinary isolates of *A. baumannii* in Croatia revealed the predominance of OXA-24-like CHDL from 2009 to 2011 [87,88,89], but it was outnumbered by OXA-23-like CHDL in later years [90]. Both OXA-23-like and OXA-24/40-like carbapenemases confer a high level of resistance to carbapenems, and the plasmids encoding CHDL usually contain resistance genes for aminoglycosides and fluoroquinolones [86,90]. The majority of isolates belonged to the widespread International clonal lineage II (IC II). OXA-24/40-like CHDL was also found in urinary *A. baumannii* isolates from Bosnia and Herzegovina [91], while OXA-58 was reported among urinary isolates from Greece linked to nosocomial outbreaks [92].

## 7. Resistance “Snapshot” in Other Notable Gram-Negative Urinary Tract Pathogens

In *P. mirabilis*, the most important resistance determinants to expanded-spectrum cephalosporins are ESBLs and pAmpC [53,93]. The most prevalent ESBL type in *P. mirabilis* is TEM-52, which efficiently hydrolyses aztreonam and ceftazime [93]; however, TEM-21 was identified among urinary isolates in France [53]. Among pAmpC, CMY-16 producing *P. mirabilis* was reported to cause outbreaks in long-term care facilities in Italy and Croatia [94,95]. In both reports, the isolates were clonally related and resistant to all β-lactams except cefepime and carbapenems. Such a trend of cephalosporinase dynamic switch from TEM variants to CTX-M and CMY was demonstrated in a more recent study from Croatia as well [96]. A study from Iraq found TEM ESBLs to be dominant among upper UTIs, with 50% positive isolates. Only 25% of the isolates harboured SHV and CTX-M beta-lactamases, respectively [40]. 

In *Enterobacter cloacae* (*E. cloacae*) isolates, a study performed in Croatia identified ESBLs belonging to CTX-M group 1 and derepressed AmpC beta-lactamases to be responsible for resistance to expanded-spectrum cephalosporins in urinary isolates [97]. CTX-M producing organisms harboured additional TEM-1 or SHV-1 beta-lactamases. ESBLs were encoded on highly transferable L/M or A/C plasmids [97]. A first report of NDM-1 in *Enterobacter aerogenes* in Croatia and Europe was also recently published [98]. A study conducted in Austria demonstrated that resistance to expanded-spectrum cephalosporins was mediated by derepressed, partially derepressed, or inducible beta-lactamases. ESBLs were found in only 4% of the isolates [99]. Urinary ESBL-positive *Enterobacter* spp. isolates were found to produce CTX-M-10 with high level cefotaxime resistance [31]. VEB-1 (Vietnam extended-spectrum beta-lactamase) was demonstrated among isolates from Thailand [69]. *bla*_VEB-1_ genes were located in class 1 integrons carrying *bla* OXA-10 and *arr* gene cassettes (the latter conferring rifampin resistance) born by self-transferable conjugative plasmids of 200 kb. Similarly, as in *E. coli*, SHV-12 was demonstrated in *Enterobacter* spp. urinary isolates from dogs in Australia. In addition to SHV-12, CMY-2 was confirmed in some of the samples [100].

As far as *Providencia* is concerned, a recent study found CTX-M beta-lactamases of group 1 among urinary *P. retgerii* and *P. stuarti* isolates from Croatia, resistant to expanded-spectrum cephalosporins [101]. These isolates were clonally related and identified during an outbreak in the University Hospital Split. *P. stuarti* from outpatients in France produced TEM-24 [53]. 

The data on antimicrobial resistance in urinary isolates of *C. freundii*, *S. marcescens,* and *M. morganii* are scarce in the medical literature. A study from Iraq found 11% of the *C. freundii* isolates from kidney infections to harbour ESBLs when compared to the isolates from the urinary bladder, which were all ESBL negative. In that study, TEM -ESBLs were dominant, followed by CTX-M and SHV-2a [40]. In urinary ESBL-positive *S. marcescens* and *M. morganii* isolates, a study from France identified TEM-24 as the dominant resistance trait [53].

## 8. Laboratory Identification of Resistance Mechanisms as a Prerequisite for Targeted Treatment

The double disk synergy test [102] and combined disk test with clavulanic acid as a confirmatory method are still pervasively used in a majority of routine laboratories to detect ESBLs in *E. coli*, *K. pneumoniae,* and *P. mirabilis* [103]. The E-test using a gradient of ceftazidime concentrations (alone and combined with clavulanic acid) and Vitek 2 are also used in some laboratories. Carbapenemases are detected by a modified Hodge test [104], CIM test [105], or CarbaNP test [105,106], depending on the laboratory protocols. The modified Hodge test has a high sensitivity in detection of KPC-producing *K. pneumoniae* and is simple to perform, thus it is often recommended as a screening test [107]; more specifically, a meropenem disk is placed on the Mueller Hinton Agar (MHA) plate, previously seeded with the 0.5 McFarland suspension of the indicator organism *E. coli* ATCC 25922 [104,108]. The isolates are then inoculated onto the plate in a straight line out from the edge of the disk to the end of the plate. Subsequently, the plates are incubated at 35 °C overnight and then growth of the indicator strain toward the carbapenem disk is examined [108]. Isolates that allowed growth of the indicator strain up to 3 mm are recorded as weakly positive, whereas those with growth of more than 3 mm were labelled positive; conversely, the absence of growth of the indicator strain toward the carbapenem disc is recorded as a negative result [108]. The CIM for the detection of suspected carbapenemase production, originally developed for *Enterobacterales*, is used for *Acinetobacter* and *Pseudomonas* as well [105]. Briefly, a meropenem 10 μg disc is incubated for two hours in a thick suspension of the tested strains, removed with a loop, and then placed on MHA inoculated with a susceptible *E. coli* indicator strain (ATCC 29522), with subsequent overnight incubation at 35 °C [105]. If a strain is a carbapenemase producer, the meropenem in the susceptibility disc is inactivated, allowing uninhibited growth of the susceptible indicator strain. The test is considered positive if the inhibition zone around the meropenem disk is less than 14 mm or if there are colonies growing inside of the inhibition zone [105]. Currently, the RESIST-4 O.K.N.V chromatographic method is available for detection of major carbapenemase types such as KPC, NDM, VIM, and OXA-48 [109]. It is easy to use, does not necessitate special equipment, and provides results in a very short amount of time with high sensitivity. Chromogenic plates to detect certain types of carbapenemases are also available, but their main drawback is a rather high cost [110]. 

Inhibitor-based tests with EDTA and phenyloboronic acid (PBA) are used to distinguish between MBLs inhibited by metal chelators and KPC inhibited by PBA. OXA-48 is detected using temocillin disks. The genes conferring resistance to beta-lactams, including broad spectrum and extended-spectrum beta-lactamases (*bla*_SHV_, *bla*_TEM_, *bla*_CTX-M,_ *bla*_OXA-9,_ *bla*_OXA-1_ and *bla*_PER-1_) [82,111,112,113], plasmid-mediated AmpC β-lactamases [114], class A (*bla*_KPC_, *bla*_SME_, *bla*_IMI_, *bla*_NMC_), class B carbapenemases (*bla*_VIM,_ *bla*_IMP_ and *bla*_NDM_), carbapenem hydrolyzing oxacillinases (*bla*_OXA-48_-like) [115], and fluoroquinolone resistance genes (*qnrA, qnrB, qnrS*) [116], can be determined by PCR, but those tests are usually only performed for research purposes.

The implementation of rapid diagnostic techniques and the development of valid prediction tools to identify ESBL infections early definitely aid in reducing the delay of the introduction of antimicrobial therapy, as well as in avoiding inadequate administration of broad-spectrum antibiotics for patients with susceptible microorganisms [117]. Consequently, several different approaches to accelerate the diagnosis of ESBLs have been explored recently, which include the detection of *bla*_ESBL_ genes by the microarray method [118] or by utilizing matrix-assisted laser desorption/ionization-time of flight (MALDI-TOF) [119,120]. Nonetheless, the aforementioned methods necessitate prior expertise and can be expensive; on top of that, as evidenced by this review, cephalosporin resistance may be caused by a plethora of genetic mechanisms that are difficult to detect by a single test. Hence, it is not likely that these methods will be introduced as a routine approach in most clinical microbiology laboratories. Regardless of the diagnostic approach pursued, the choice of the UTI treatment depends on the type of infection, causative agents, in vitro susceptibility, age of the patient, and the general condition of the patient in question.

## 9. Conclusions

This review clearly showed that there is a high variety of different resistance mechanisms among urinary isolates (summarized in Table 1), while the prevalence patterns of different resistance mechanisms are highly dependent on the local epidemiology. Although this leaves us with an interesting global resistance map, it has to be taken into account that studies were conducted in different time periods. However, the fact remains that due to an increase in the prevalence of multidrug or extensively drug-resistant strains among urinary tract Gram-negative pathogens, as well as the lack of new antibiotics, the therapeutic options are getting severely limited. Therefore, there should not only be increased efforts and research projects focused on innovations in the field of antimicrobial stewardship in ambulatory settings, but also on its impact on the emergence and spread of ESBL-producing microorganisms in community settings.

## Figures and Tables

**Table 1:** A literature-informed summary of resistance mechanisms described in Gram-negative urinary isolates.

	ESBL	AmpC	Carbapenemases	Aminoglycoside Resistance Mechanisms	Fluoroquinolone Resistance Mechanisms	References
						(Sorted Alphabetically)
*Escherichia Coli*	CTX-M-1 CTX-M-3 CTX-M-14 CTX-M-9 CTX-M-15 CTX-M-27 CTX-M-30 SHV-7 OXA-1 TEM-21 TEM-22 TEM-28 IRT-3 SHV-12	CMY-2	KPC VIM IMP OXA-48 Efflux pumps	*aacC2*	*qnrA*, *qnrB*, *qnrS*, mutations in *gyrA* and *parC* genes	Arpin, 2003 Blazquez, 1993 Bohnert, 2006 Bou, 2002 Bradford, 1996 Kim, 2020 Onanuga, 2019 Rodrigez-Bano, 2004 Teshager, 2000 Yasufuku, 2011
*Klebsiella Pneumoniae*	CTX-M-15 SHV-2 SHV-2a SHV-5 TEM-15 TEM-19 TEM-21 TEM-24 TEM-48	DHA-1 FOX CMY-2	KPC VIM IMP OXA-48	16S rRNA methylases: *rmtA, rmtB*, *armA* *aadB* *aadA2*	*qnrA*, *qnrB*, *qnrS*, mutations in *gyrA* and *parC* genes	Arpin, 2003 Bedenić, 1998 Bedenić, 2001 Bedenić, 2010 Damjanova, 2007 Damjanova, 2008 Gniadkowski, 1998 Gruteke, 2003 M’Zali, 1995 Podbielski, 1991 Thomson, 1991 Vedet, 2006 Zujić-Atalić, 2014
*Enterobacter* spp.	CTX-M-15 CTX-M-10 VEB-1	Derepressed AmpC	VIM-1			Apfalter, 2002 Franolić-Kukina, 2016 Girlich, 2001
*Proteus* spp.	TEM-52	CMY-16				Bedenić, 2016
						Sardelić, 2010
*Klebsiella Aerogenes*	TEM-3 TEM-24					Arpin, 2003
*Citrobacter* spp.	CTX-M-2 CTX-M-14 CTX-M-15 SHV-12				qnrB4 qnrS AAc6-Ib-cr	Kanamori, 2011
*Acinetobacter Baumannii*			OXA-23 OXA-24 OXA-58	16S rRNA methylases: *armA*, *rmtB*	mutations in *gyrA* and *parC* genes	Franolić-Kukina, 2011 Garneau-Tsodikova, 2016 Ladavac, 2015 Pournaras, 2006 Vranić-Ladavac, 2014
*Pseudomonas* *Aeruginosa*	OXA-10 OXA-17 SHV -5		VIM-2	16S rRN Amethylases: *armA, rmtA, rmtB, rmtD1, rmtG* *aacC6*	mutations in *gyrA* and *parC* genes	Bošnjak, 2010 Bubonja-Šonje, 2015 Garneau-Tsodikova, 2016 Poirel, 2004 Sardelić, 2012 Sorour, 2008

## Data Availability

Not applicable.
